# ^99m^Tc-MDP bone scintigraphy of the hand: comparing the use of novel cadmium zinc telluride (CZT) and routine NaI(Tl) detectors

**DOI:** 10.1186/s13550-015-0139-6

**Published:** 2015-11-14

**Authors:** Victoria Koulikov, Hedva Lerman, Mikhail Kesler, Einat Even-Sapir

**Affiliations:** Department of Nuclear Medicine, Tel-Aviv Sourasky Medical Center, Sackler Faculty of Medicine, Tel-Aviv University, 6 Weizman Street, Tel-Aviv, 64239 Israel

**Keywords:** Cadmium zinc telluride (CZT), Detectors, Bone, Hands

## Abstract

**Background:**

Cadmium zinc telluride (CZT) solid-state detectors have been recently introduced in the field of nuclear medicine in cardiology and breast imaging. The aim of the current study was to evaluate the performance of the novel detectors (CZT) compared to that of the routine NaI(Tl) in bone scintigraphy. A dual-headed CZT-based camera dedicated originally to breast imaging has been used, and in view of the limited size of the detectors, the hands were chosen as the organ for assessment. This is a clinical study.

**Methods:**

Fifty-eight consecutive patients (total 116 hands) referred for bone scan for suspected hand pathology gave their informed consent to have two acquisitions, using the routine camera and the CZT-based camera. The latter was divided into full-dose full-acquisition time (FD CZT) and reduced-dose short-acquisition time (RD CZT) on CZT technology, so three image sets were available for analysis. Data analysis included comparing the detection of hot lesions and identification of the metacarpophalangeal, proximal interphalangeal, and distal interphalangeal joints.

**Results:**

A total of 69 hot lesions were detected on the CZT image sets; of these, 61 were identified as focal sites of uptake on NaI(Tl) data. On FD CZT data, 385 joints were identified compared to 168 on NaI(Tl) data (*p* < 0.001). There was no statistically significant difference in delineation of joints between FD and RD CZT data as the latter identified 383 joints.

**Conclusions:**

Bone scintigraphy using a CZT-based gamma camera is associated with improved lesion detection and anatomic definition. The superior physical characteristics of this technique raised a potential reduction in administered dose and/or acquisition time without compromising image quality.

## Background

Gamma cameras are composed of semiconductor cadmium zinc telluride (CZT) detectors dedicated to cardiac perfusion studies and to molecular breast imaging (MBI). Cardiac CZT-based cameras were found to offer ultrafast myocardial perfusion imaging with better spatial resolution and count rate, thus allowing reduction of injected dose and/or acquisition time potentially overcoming the problems of motion artifacts and improved patient comfort [1–8]. Dual-headed CZT-based cameras dedicated to MBI have been shown to be valuable in detection of small lesions of clinical relevance [9–14]. The latter system has been installed in our facilities and is being used for detection of breast pathology. The detection of small breast lesions encouraged us to conduct the current study and assess the role of the CZT-based camera for detection of bone and joint pathology on ^99m^Tc-methylene diphosphonate (MDP) bone scintigraphy. In view of the small size of the field of view, imaging of the hands was chosen as our model.

## Methods

### Patient population

The study cohort consisted of 116 hands of 58 consecutive patients, 30 male and 28 female, age 17–90 years (mean 42 ± 10), referred for ^99m^Tc-MDP bone scintigraphy for suspected hand pathology. Indications for scintigraphy included trauma (*n* = 26), pain (*n* = 28), unclear lesions on radiography (*n* = 2), suspected reflex sympathetic dystrophy (RSD) (*n* = 1), and sarcoma (*n* = 1).

The study was approved by the ethical committee of the Tel Aviv Sourasky Medical Center.

### Bone scintigraphy

**Fig. 1:**
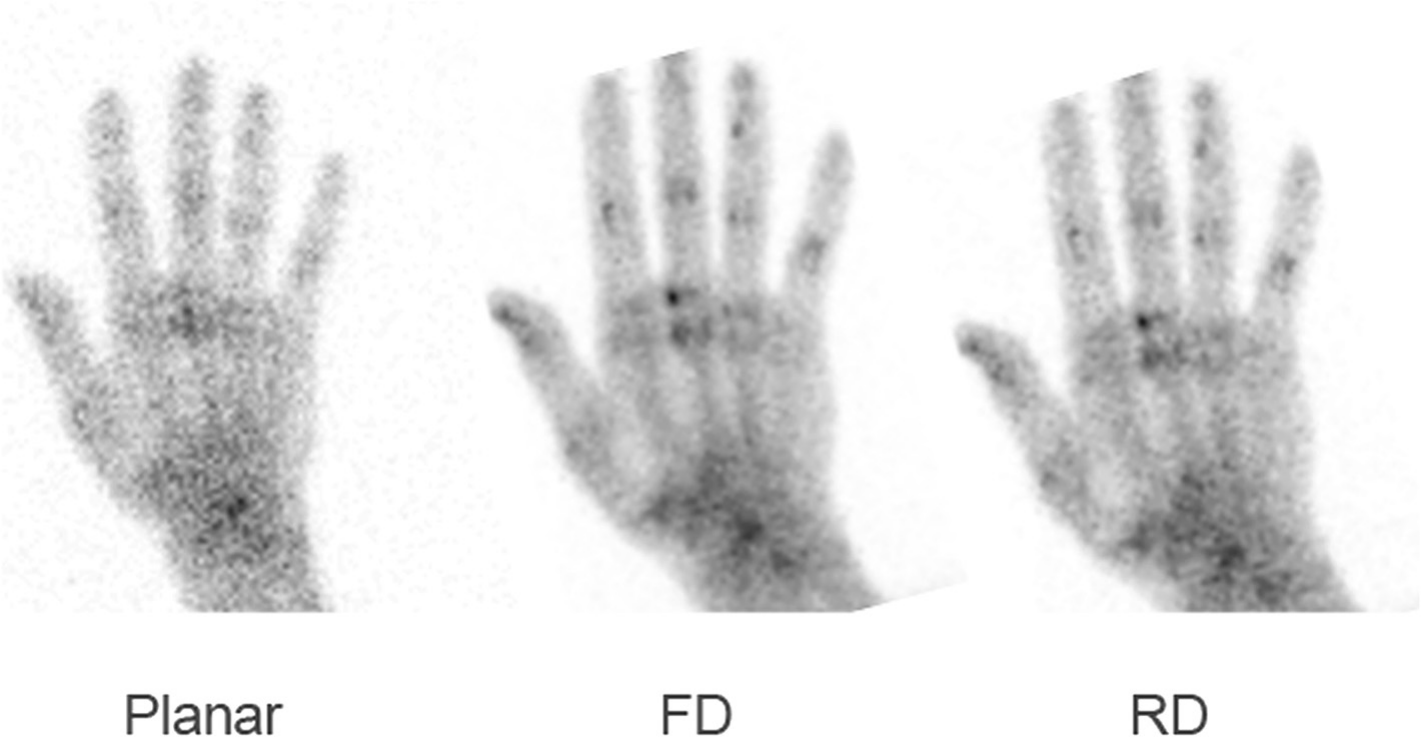
A 74-year-old man with non-specific pain in the skeleton including the hands. From *left* to *right*: routine NaI(Tl) camera, full-dose (*FD*) CZT detector, and reduced-dose (*RD*) CZT detector image sets. Note the anatomic details of the bones (including the sesamoid bones) and joints on the CZT data, either FD or RD

Two to three hours after the injection of 740 MBq ^99m^Tc-MDP, data acquisition was performed using two separate systems: a routine gamma camera (Infinia or Optima, GE Healthcare) with a high-resolution collimator and a CZT-based camera (Discovery NM750b, GE Healthcare) originally aimed for breast imaging. Using the routine camera, the hands were placed on one of the camera heads for 10 min. For acquisition using the CZT-based camera, the hands were placed on the lower detector while the upper detector was used to immobilize the hands without pressure. Acquisition on the CZT-based camera was in dynamic mode, ten images of 1 min each. The combined data of all ten images reflect the full-dose full-acquisition time (full data, FD). A second set of images combining data of the first 4 min only (reduced data, RD) were generated reflecting the images obtained by a short (4 min) acquisition or simulating a reduced (296 MBq) injected dose. Thus, for each patient, three image sets of the hands were available for assessment: a set obtained by routine camera, FD, and RD data (Fig. 1).

### Data analysis

Images were interpreted by two nuclear medicine experts (VK, HL) in consensus reading. The three data sets were reviewed at least 2 weeks apart, with the readers blinded to the patient’s name and clinical data. For assessment of lesion detection, any site of increased uptake was recorded. Interpretation of increased uptake was based on comparison with the contralateral bone or with the adjacent bone if both hands showed abnormality.

**Fig. 2:**
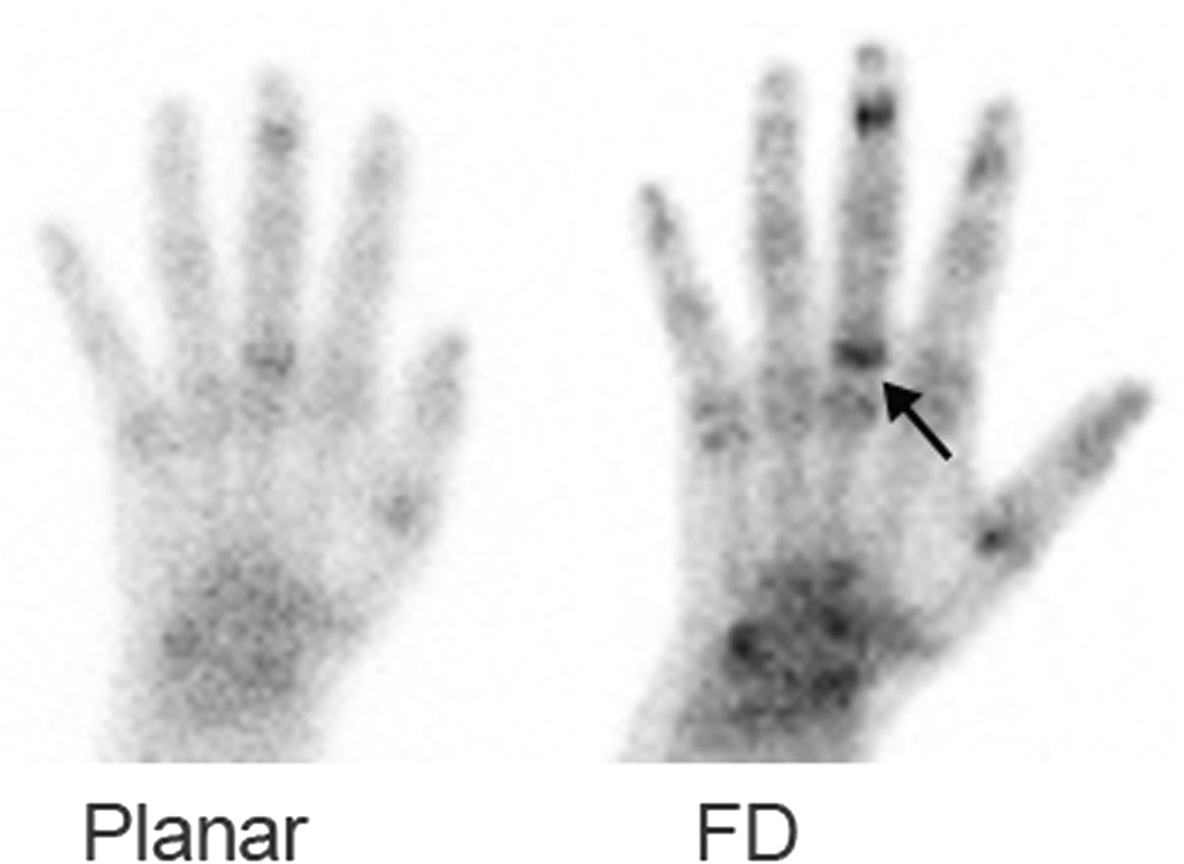
A 72-year-old female with non-specific pain in the hands. Note on the CZT data the detection of a joint space of the third metacarpophalangeal joint. Increased uptake is seen only at the bone distal to the joint space (*arrow*)

The criterion for assessment of image quality was the ability to visualize the location of the metacarpophalangeal (MCP), proximal interphalangeal (PIP), and distal interphalangeal (DIP) joints. Experts also commented on the ability to identify the joint space, i.e., to visualize the cold area representing the joint space separating the proximal and distal bones. On the CZT data of Fig. 2, for instance, the joint space of the MCP joint of the third finger is clearly seen while the location of the DIP joint of this finger can be identified but the joint space of this joint is not visualized. This figure also illustrates the ability to determine on the CZT data whether abnormality is present in the bone on both sides or only at one side of the joint space.

The ability to determine the joints’ location on the data sets was compared using the McNemar test with *p* < 0.05 considered statistically significant.

## Results

**Fig. 3:**
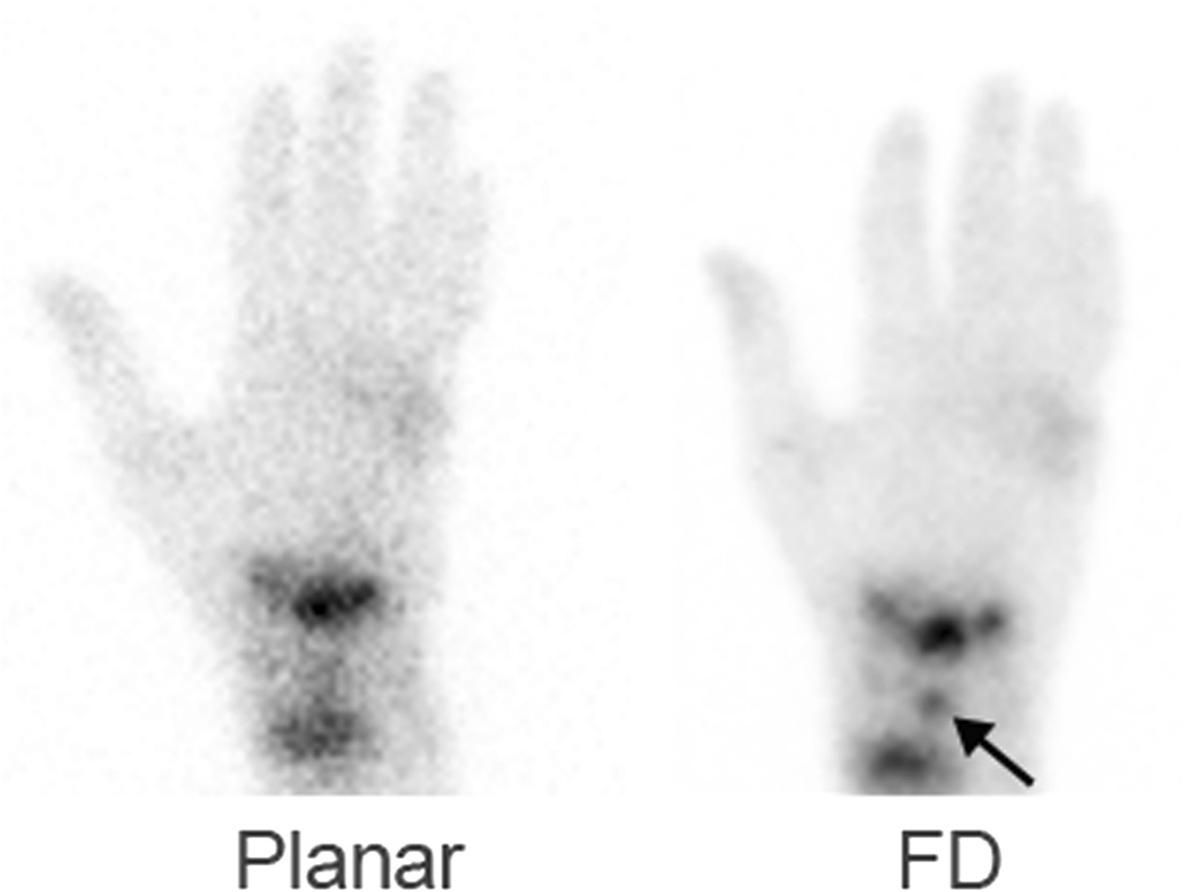
A 25-year-old female post left hand trauma. Bone scintigraphy acquisition took place while the hand was in a cast. In addition to the focal increased uptake identified at the distal radius and distal carpal bone, focal uptake is identified in the lunate bone only on the CZT data (*arrow*)

**Fig. 4:**
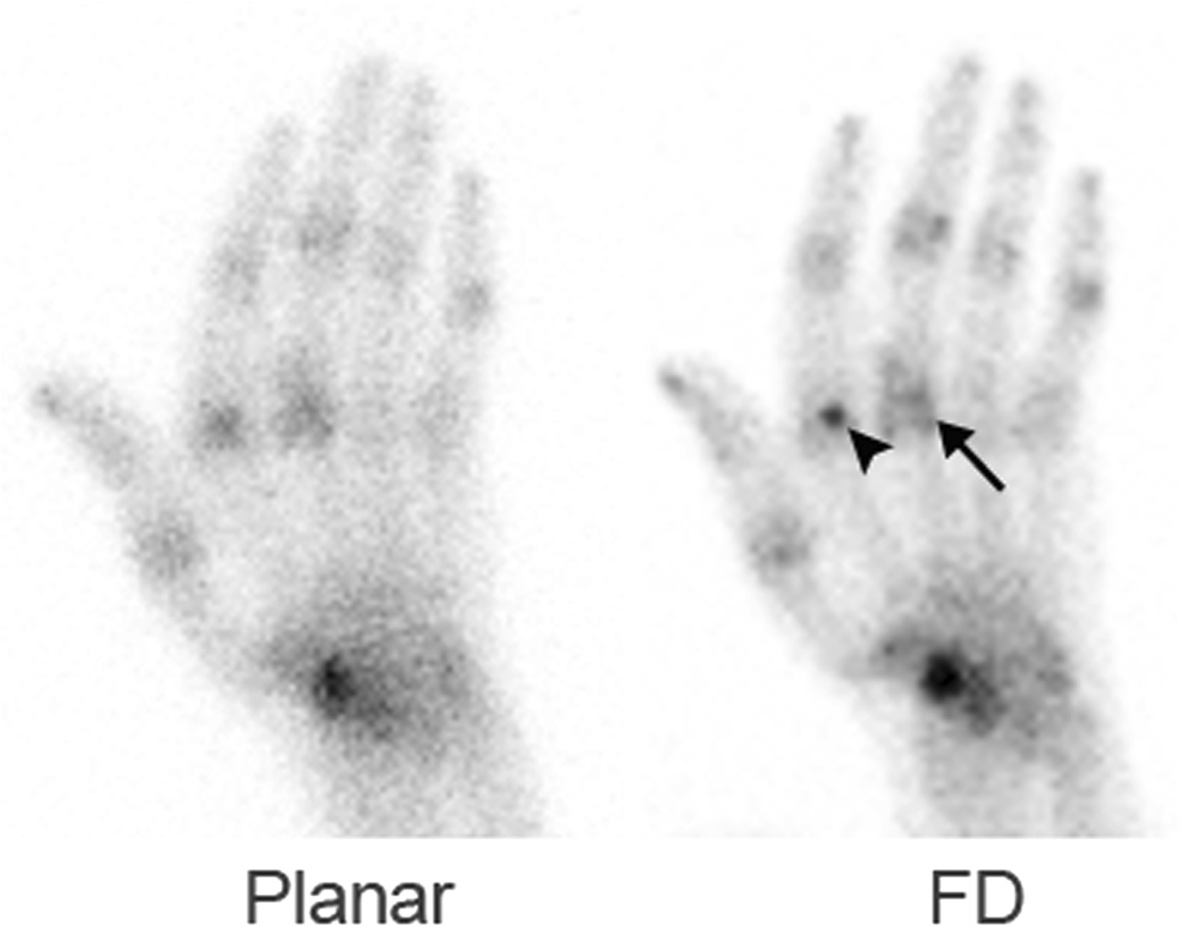
A 71-year-old man with rheumatoid arthritis. On both image data, multiple joints appear to be associated with increased uptake. On the CZT data, uptake is located on both sides of the joint space (*arrow*) and the increased uptake at the metacarpophalangeal region of the index finger appears to be a sesamoid bone (*arrowhead*)

Sixty-nine hot lesions were identified on CZT data whereas only 61 focal sites of increased uptake were recorded on NaI(Tl) images (no statistically significant difference) (Figs. 3 and 4).

Table 1 summarizes the visualization of the MCP, PIP, and DIP joints by each of the three image sets. Of the 1624 joints present in the 116 evaluated hands, locations of 168 joints were identified using routine detectors compared to 385 joints when using FD CZT (sensitivity 0.1 compared to 0.24, *p* < 0.001, 95 % CI 0.145–0.186). No difference was found between FD and RD images as the latter identified 383 joints. Figures 3 and 4 demonstrate the better detection of the joints on the CZT data, identifying also whether increased uptake is present in the bone on both sides or only at one side of the articulation.

**Table 1:** Visualization of joints on three image sets: using the routine NaI(Tl) camera, using full-dose full-time acquisition with CZT detectors (FD CZT), and using reduced-dose reduced-time acquisition with CZT detectors (RD CZT)

Image set	Total number of lesions	MCP	PIP	DIP
Standard camera	168	116	47	5
FD CZT	385	219	137	29
RD CZT	383	219	137	27

*MCP* metacarpophalangeal joint, *PIP* proximal interphalangeal joint, *DIP* distal interphalangeal joint

## Discussion

Novel gamma cameras with CZT detectors have been recently introduced in the routine of nuclear medicine, and currently, there are systems dedicated to molecular breast imaging (MBI) and cardiac perfusion imaging. The novel geometric design and new detector material combined with new reconstruction algorithms have been shown in the literature to improve the diagnostic performance of gamma cameras in cardiac perfusion studies and scintimammography with superior image quality, shorter acquisition times, and reduced radiation exposure [1–14].

Bone scintigraphy is the most common procedure in nuclear medicine departments that plays an important role in the diagnosis of various skeletal disorders, including trauma, joint disease, infection, inflammation, and neoplastic conditions. The role of CZT detectors for bone scintigraphy has not been fully appreciated. As the CZT camera available in our facility is that composed of a 20 × 20 cm^2^ detection area tailored from breast imaging, we chose a small organ of interest, the hands, to explore the potential benefit of CZT detectors in bone scintigraphy. Bone scintigraphy is a common procedure for assessment of bone and joint pathology in the hand allowing the detection of posttraumatic changes, infection, inflammatory or degenerative changes, avascular necrosis, reflex sympathetic dystrophy, and neoplastic processes. Bone scintigraphy of the peripheral skeleton mainly of the small bones and joints of the hands burdens a potential suboptimal spatial resolution and poor anatomical definition often requiring a longer acquisition time, zoomed acquisition, and even change of collimators. Image quality may be even more deteriorated in patients with poor blood perfusion secondary to cardiovascular disease or peripheral vascular disease, who are overweight, or with renal and metabolic diseases resulting in a decreased tracer dose in the extremities. In children, the bones and joints composing the hands are of particular small size and there should be an ongoing effort to reduce administered dose and radiation exposure.

Evaluation of arthritis is an ongoing imaging challenge. Scintigraphy using ^99m^Tc-MDP, labeled immunoglobulin G (IgG) gamma imaging, and ^18^F-FDG PET are used for joint imaging [15–20]. Improved image quality and visualization of the hand joints was achieved by using a positron emission mammography (PEM) scanner (arthro-PEM) [21]. The detection of active inflammation using non-specific tracers is primarily based on non-specific mechanisms such as hyperemia and increased vascular permeability. Recently, specific radiolabeled mAb including anti-CD20 and anti-TNF-α have been introduced for imaging of chronic inflammatory autoimmune joint diseases such as rheumatoid arthritis. In addition to evaluation of disease extent and severity, findings of immunoscintigraphy may assist in selection of patients that could benefit from immunotherapy and for monitoring response to therapy, maintaining the approach of personalized medicine [22–26]. The results of our study indicate that image quality reflected by visualization of joints is significantly improved with the CZT detectors compared to imaging using the routine NaI(Tl)-based camera, occasionally allowing to define whether the scintigraphic abnormality is one sided or present on both sides of the articulation. These results raise a potential improved performance of immunoscintigraphy of the joints using a CZT-based device. However, at present, the commercially available CZT systems are organ specific and/or small while imaging of joint diseases warrants imaging of larger joints and a whole-body approach.

In the current study, we did not find a significant difference neither in lesion detection nor in image quality between full-dose data and data that simulated reduced acquisition time or reduced injected dose. This is a major potential benefit of CZT detectors in the clinical practice of nuclear medicine.

There are several limitations to the study. First is the inherent lack of a gold standard for the final diagnosis of the abnormal scintigraphic findings. The other is the variable indications of bone scan of the hands and therefore the heterogeneous study cohort. This is however a pilot study, and its results encourage further evaluation of the role of CZT technology in specific indications of bone scan as well as scintigraphy using other tracers.

## Conclusions

Bone scintigraphy with the novel CZT detectors is associated with improved image quality. Radiation dose and/or acquisition time can be considerably reduced without compromising lesion detection or image quality using this technology.
